# The Rationality of Prejudices

**DOI:** 10.1371/journal.pone.0030902

**Published:** 2012-02-16

**Authors:** Thomas Chadefaux, Dirk Helbing

**Affiliations:** Chair of Sociology, in particular of Modeling and Simulation, Swiss Federal Institute of Technology Zurich, Zurich, Switzerland; Hungarian Academy of Sciences, Hungary

## Abstract

We model an 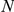-player repeated prisoner's dilemma in which players are given traits (e.g., height, age, wealth) which, we assume, affect their behavior. The relationship between traits and behavior is unknown to other players. We then analyze the performance of “prejudiced” strategies—strategies that draw inferences based on the observation of some or all of these traits, and extrapolate the inferred behavior to other carriers of these traits. Such prejudiced strategies have the advantage of learning rapidly, and hence of being well adapted to rapidly changing conditions that might result, for example, from high migration or birth rates. We find that they perform remarkably well, and even systematically outperform both Tit-For-Tat and ALLD when the population changes rapidly.

## Introduction

People frequently judge and discriminate each other on the basis of their skin color, gender, or clothing style. Beyond the ethical issues they raise, such prejudices seem inefficient. They often lead to erroneous judgements, missed opportunities and resentment and, at the aggregate level, to segregation, riots, or religious conflicts [1]. Why, then, are they so ubiquitous?

In this paper, we argue that prejudices strive because strategies that rely on them can be very successful in competitive environments. Prejudices are heuristics based on accumulated experiences, expressed as simple cognitive relationships between specific traits (e.g., height) and behavior (e.g., aggressiveness). These associations, acquired through evolution or experience, enable people to reach judgments about complex situations or competitors in a “blink” [2]–[7]. In this sense, they are closely related to people's ability to process large amounts of information rapidly and often unconsciously, and to reach quick decisions [8]–[10].

One explanation for these “heuristics that make us smart” [9] is evolutionary. When our ancestors interacted with strangers, those who could rapidly and accurately discriminate between dangerous and trustworthy partners were more likely to survive and reproduce. However, this explanation does not inform us about the conditions under which relying on these cognitive shortcuts is rational or optimal [11]. The fact that, empirically, people do rely on rules of thumb certainly implies that these rules can be useful and efficient, but not that they always are. In fact, even though our intuitive judgments are often accurate, they also frequently lead to errors and inaccuracies [12], so that the opposite argument could be made equally well: evolutionarily, those most likely to survive are those able to assess situations calmly and to derive rational conclusions–not hasty responses based on emotions or “gut-feelings” [13], [14].

In summary, we know little about the fitness of strategies that use only a limited subset of the available information to reach conclusions about their social partners. Can prejudices–the extrapolation onto others of the behavior of people characterized by similar attributes–form the basis of a successful strategy in a competitive environment? In this paper, we investigate the performance of these rules of thumb by putting them in competition with well-known strategies such as Tit-For-Tat or defection. We derive conditions under which prejudiced strategies outperform these other strategies and when, on the contrary, they are suboptimal.

Prejudices have the advantage of providing pre-defined guidelines for interactions, without the need to learn the other's specificities. As such, they enable rapid reactions to unknown circumstances–for example those involving a significant portion of interactions with foreigners. We will show that they can successfully avoid exploitation, while still taking advantage of cooperation with populations that have been found to be cooperative. Of course, such learning speed comes at a cost. Because prejudices are coarse-grained inferences based on a limited number of attributes, they are particularly prone to error (e.g., not all green people are uneducated) and hence lead to some level of exploitation (e.g., wrongly assuming that all blue people are cooperators) or missed opportunities.

Despite this inaccuracy, we find that strategies based on prejudices perform well for a large range of parameters. They are particularly well suited for situations in which the population renews itself relatively rapidly–for example because of high migration or birth rates. In these situations, they even outperform the most successful strategies that have previously been proposed in the literature [15]: those based on reciprocity (Tit-For-Tat, a strategy in which a player starts cooperating, and then copies the interaction partner's decisions), and those based on exploitation (ALLD, in which an individual always defects).

## Methods

To model prejudices, we assume that individuals are diverse [16], [17]. They are defined by observable attributes (e.g., age, gender, or wealth) which affect–albeit to different degrees–their propensity to follow one strategy or another. For example, blue, short and young people might be more likely to cooperate, whereas green, tall and old ones are more likely to defect. These relations are unknown to individuals in the beginning. Instead, players must rely on their experience–previous interactions with other people exhibiting these traits–in order to extrapolate the effect of certain traits on behavior–the more traits, the more difficult inferences are.

The idea that behavior and certain visible traits are correlated may not always be accurate. Still, we believe that many observable cues (e.g., age, displays of wealth) can provide at least some information about people's capabilities and probable behavior. A person's height, for example, appears to be correlated with her ability and job market performance [18]. We are not arguing here that observable traits are sufficient to determine behavior, but rather that there exists a correlation that can be uncovered. We explore later the impact of watering down that correlation by adding noise in the mapping from attributes to strategies.

In our model, players draw inferences differently depending on their level of prejudice. Highly prejudiced individuals focus on only one attribute (e.g., height), and base their behavior on this trait alone (e.g., tall people tend to be aggressive, hence I should defect). Less prejudiced individuals, on the other hand, will be more nuanced and base their decisions on additional attributes (e.g., I know that tall, blue and poor people tend to be cooperative, but I cannot judge on the basis of height alone). In this sense, Tit-For-Tat–responding in kind to the opponent's previous actions–is the most unprejudiced strategy, in that it treats every individual as unique and does not make any assumption about others, even if they carry the same traits as those of previously encountered individuals.

More formally, individuals denoted by 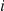 are assigned a random vector of 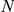 traits (analogous to a “DNA sequence”) 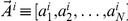, where 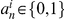 is a binary attribute that can be interpreted as, for example, player 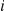
's color (red/blue), gender (male/female), or clothing (rich/poor).

Players can have one of three possible strategies: Tit-for-tat (TFT), whereby the player cooperates in the first round, after which she copies what her partner did in the previous round; Always Defect (ALLD), whereby the player always defects, regardless of the history of play; and Prejudiced, whereby a player bases its response on the traits of her opponent.

Prejudiced players, denoted by 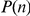, observe the first 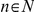 of their interaction partners' attributes and, on that basis, decide whether to cooperate. We call those with a small 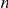 highly prejudiced players, and those with a large 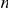 little prejudiced players. For example, a highly prejudiced player, 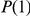, would observe only the first attribute (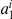) of its partners' DNA sequence 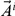, whereas a less prejudiced one, 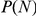, would observe all 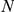 traits.

**Figure 1:**
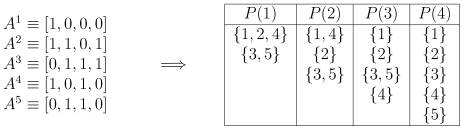
Illustration of the classifications made by a prejudiced individual as a function of her level of prejudices. On the left, we show the DNA sequence of five players, and on the right, the way these players would be categorized by a prejudiced individual according to its level of prejudice. A very prejudiced player, 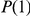, only observes the first attribute, and hence forms coarse groups. 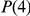, on the other hand, observes all four attributes and, since no two individuals share the same DNA, treats each individual as unique.

To illustrate the idea, consider a sample population of 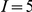 individuals with 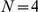 traits, and how they would be categorized by prejudiced players (Fig. 1). 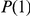 only observes and bases its response strategy on attribute 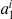, and hence forms very coarse groups (e.g., green vs. blue people). On the contrary, 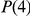 draws no inference, since no two individuals share the same sequence of traits. In other words, it treats each individual as unique in this example. Clearly, observing more attributes before reaching an opinion contributes to a finer-grained view of the population, because it splits it into smaller and smaller subsets. However, it has the disadvantage of requiring the observation of a larger population before inferences can be drawn.

Based on these observations, 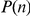 forms beliefs 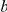 about specific sequences of traits. A belief is a mapping 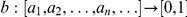. That is, 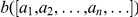 denotes 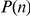
's belief about the propensity of players with attributes 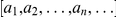 to cooperate. We assume that 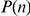 initially believes that all players are cooperators with probability one. This belief is updated after every interaction as a function of the other player's behavior: the belief is simply the average of the actions of those individuals of a specific group that 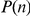 has met. If, for example, 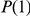 has met three players with 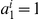, of which two have defected and one has cooperated, then 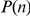
's belief about individuals with 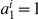 is that they defect with probability 2/3, and hence 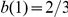. 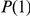 then cooperates with the next individual exhibiting 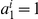 with probability 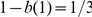, and updates its beliefs again as a function of what that new opponent does. In a sense, then, all individuals with the same first 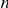 traits are treated as if they were the same person (we investigate later the consequences of potential misperceptions–e.g., seeing a blue person as red). More complex learning strategies (e.g., Bayesian updating) could easily be implemented, but our point here is that even the most basic learning algorithm is sufficient for our results.

At the beginning of the game, each player's strategy 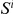 is defined as follows:
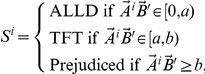
where 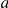 and 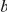 are simple parameters that affect the proportion of each strategy in the population, and 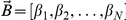 (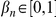 and 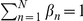) is a random vector of weights. Note that we assume that 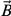 is a random vector, but that it is common to all players. This assumption is crucial, because it implies that strategies are, to some extent, determined by the players' attributes. In other words, a correlation between attributes and behavior is assumed to exist, although it is initially unknown to the players. We investigate below the effect of loosening that correlation by incorporating various types of “noise.”

The game proceeds in 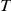 steps. In each of them, players are randomly paired to play a prisoner's dilemma with another player. The payoffs are simply the ones used in Axelrod's original tournament [15]:
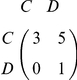
At the end of the game, each player's average payoff is calculated and used as measure of its “fitness” or success. Note that, to keep things simple and to ensure that no other mechanism is causing our result, we assume that there is no imitation involved: players follow one strategy and never deviate from it (however, we investigate the role of noise below).

To sum up, we model an 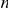-player repeated prisoner's dilemma game in which players are randomly assigned a vector of attributes. These attributes determine their strategy (TFT, ALLD or prejudiced) according to a function unknown to the players, but common to all of them. Prejudiced players observe one or more of their partners' attributes (P(1) observes the first one, P(3) the first three, etc.), and draw inferences on the basis of their interactions. These inferences then form the basis of the prejudiced player's probability to cooperate or defect with future partners.

## Results

We find that the performance of various strategies depends fundamentally on the shadow of the future [15]–the expected number of interactions that a player has with a given individual over the course of the entire game. More specifically, the shadow of the future is defined as 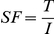, where 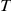 is the duration of the game and 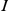 is the total number of players. Note that 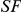 refers to an *expected* number of interactions, since partners are chosen randomly in each time step. A long shadow of the future does not guarantee that two players will meet several times over the course of a game, but simply that there is a high probability that they will.

**Figure 2:**
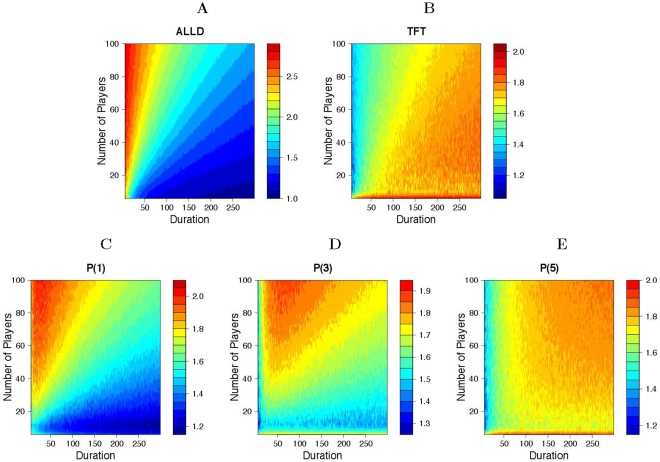
Performance of prejudiced and non-prejudiced strategies. Average score of different strategies as a function of the two components of the “shadow of the future” 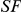 (game duration
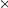
number of players). The five “heat” plots illustrate the average payoff of various strategies in a population split equally between TFT and ALLD players (A and B). In simulations C–E, one single prejudiced player is added to each simulation. Each individual is given five random binary traits, and prejudiced players extrapolate based on one (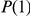), three (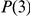) or all of these traits (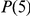). TFT performs well when the shadow of the future is long (small population and long duration). In contrast, ALLD performs best for a short shadow of the future. Prejudiced strategies perform best for intermediate shadows of the future. Each data point on the 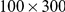 grid is an average over five hundred simulations, for a total of 15 million simulations.

For most of our results, we consider a simulation in which each player has a total of five attributes (we also study below the impact of varying the total number of attributes). In line with [19], we find that ALLD does best for a short shadow of the future (a low duration combined with many players), because defection is then clearly the dominant strategy (Fig. 2 A). TFT, on the other hand, does not draw inferences, and hence exposes itself to exploitation every time it encounters a new partner. This risk of exploitation pays off in the long run because it allows TFT players to accurately learn the other players' strategies, but not if the shadow of the future is short (Fig. 2 B). As an example, consider a situation with 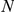 players and duration 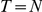. In such a situation, the shadow of the future is 1, i.e., each player expects to meet every other individual only once over the course of the game. Assume moreover that all players follow the strategy ALLD. In such a world, a TFT player would clearly do poorly, since it would cooperate with–and be exploited by–every player. By the end of the game, it would know precisely who is a defector (everyone) with certainty, but this knowledge would be useless at this point, since the short shadow of the future implies that it most likely will never meet them again. This is what we mean when we say that TFT learns precisely, but slowly.

On the contrary, a prejudiced player would perform quite well in such a world. It would start by cooperating in its first few interactions, but quickly draw inferences about the rest of the population, and hence avoid exploitation even against partners it has not yet met. However, while extrapolation allows prejudiced players to be fast at identifying patterns (e.g., “blue people tend to defect”) and, as a result, to avoid exploitation on a large scale, it also implies a potentially high error rate: missed opportunities to cooperate, or defection against cooperators. In contrast, less prejudiced strategies (TFT being an extreme case) reach a very accurate picture of each individual's behavior, but obtaining this picture is slow and hence subject to initial exploitation.

This trade-off between expediency and precision is visible even between different levels of prejudices. Thus, the strategy 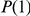, which only observes one of the five attributes, learns very rapidly, and hence does well when the shadow of the future is relatively short (Fig. 2 C). However, because it misjudges its opponents too frequently, it also has a high error rate, and therefore performs poorly when the shadow of the future is long. 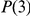, a strategy in which 3 of the 5 traits are observed, is an intermediate case between 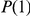 and 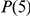: it is slower to learn than 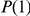, but also makes fewer mistakes (see Fig. 2 D). Finally, 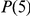 observes all 5 attributes and is quite slow to learn–however, it is still much faster than TFT because it extrapolates–and hence performs relatively poorly when the shadow of the future is short, but well when it is long (Fig. 2 E). Note in particular the similarity with TFT, for which every player is treated as unique.

**Figure 3:**
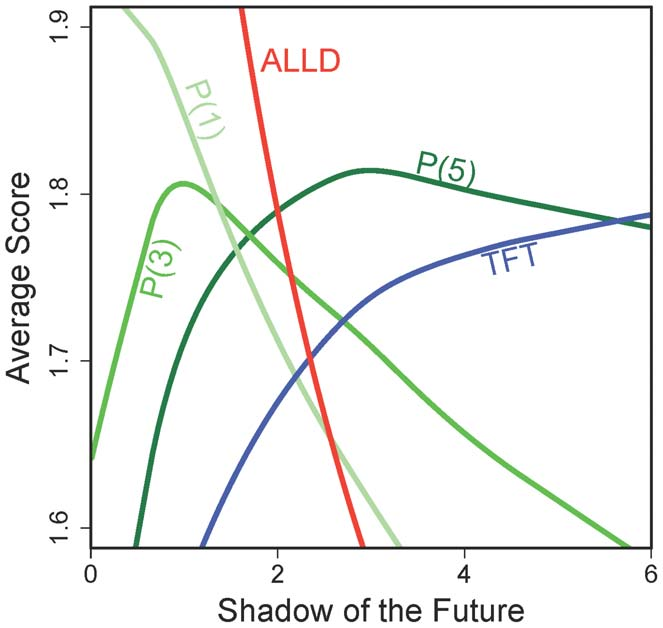
Prejudiced strategies outperform TFT and ALLD for intermediate shadows of the future. Average score of various strategies as a function of the shadow of the future. TFT performs best when the horizon is long. ALLD, on the other hand, performs best when the horizon is short. For any intermediate shadow of the future, prejudiced strategies (such as P(5)) obtain higher average scores than either ALLD or TFT. The results are based on 100 simulations with 50% TFT, 50% ALLD and one prejudiced player.

Comparing the relative performances of these different strategies confirms this finding, and also demonstrates the superiority of prejudiced strategies for intermediate values of the shadow of the future (Fig. 3). In particular, both 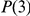 and 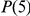 manage to outperform both ALLD and TFT for shadows of the future between 2 and 6 (a range that includes a large portion of real-world interactions).

**Figure 4:**
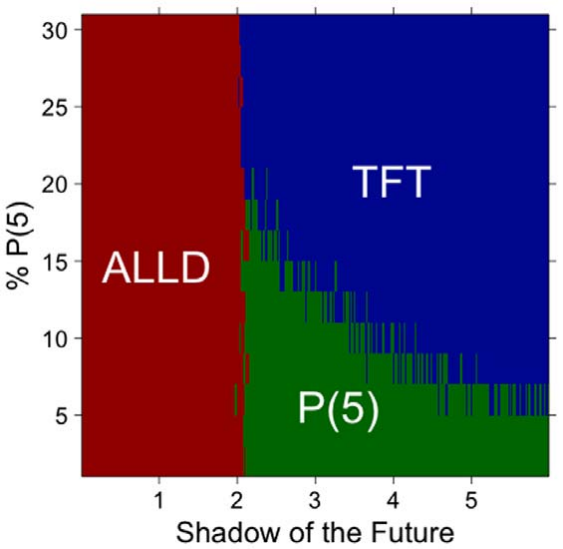
Strategies with the highest average score, as a function of the proportion of prejudiced players. Each individual is given five traits; the proportion of prejudiced (P(5)) players added to the simulation is “% P(5)”, with the remaining population equally split between ALLD and TFT players. For short shadows of the future, ALLD defeats all other strategies. For longer shadows of the future, prejudiced players can beat TFT, but only if the total proportion of prejudiced players in the population remains sufficiently low. In other words, prejudiced strategies perform well against TFT and ALLD, but not against themselves.

We have shown that prejudiced strategies perform well against both TFT and ALLD. But how do they perform against themselves, i.e., against other prejudiced players? To answer this question, we varied the proportion of the population that relies on prejudiced strategies, and found that the more prejudiced players are added to the simulation, the lower their performance becomes. In other words, prejudiced players perform best in isolation (Fig. 4). There are at least two reasons for this result. First, a large proportion of prejudiced players imply a higher number of missed opportunities. This is because prejudiced players rely on crude approximations, and hence two of them interacting is likely to multiply the probability with which at least one misestimates the other. Second, and most important, prejudiced strategies are inconstant because prejudiced players cooperate or defect based on their (evolving) prejudices–not on their genetic encoding. Prejudiced strategies strive among players whose strategies are relatively stable over time, but are error-prone against inconstant ones. As a result, inferences drawn about their strategy at time 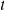 are unlikely to be correct at time 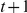. Prejudiced players are good at simplifying the world, but fail when the world is too complex.

**Figure 5:**
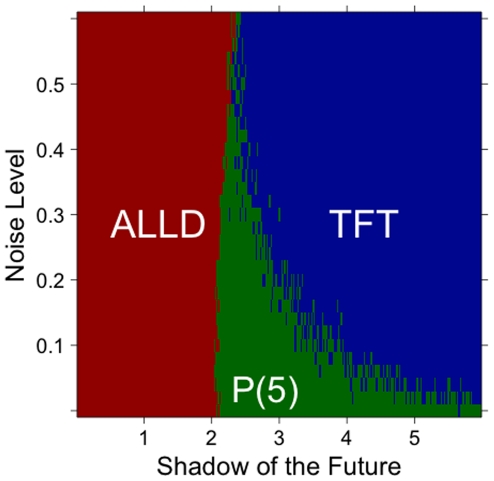
Strategies with the highest average score, as a function of the level of noise. Noise refers to the probability that a player's strategy does not correspond to its DNA. In other words, it weakens the correlation between specific traits and strategy. The higher the level of noise, the more players' strategies are defined independently of their traits. Prejudiced strategies perform well for low levels of noise and intermediate values of the shadow of the future. For high levels of noise, however, inferences drawn by prejudiced strategies become less and less reliable. The results are based on one hundred simulations with 50% TFT, 50% ALLD and one prejudiced player.

In addition, we investigated the performance of these various strategies in the presence of different types of noise [20]. We expect noise to be particularly problematic for prejudiced strategies, because it makes learning far more difficult. First, we assumed that the mapping from traits to strategy can be imperfect, in the sense that “DNA” might not determine behavior. More specifically, we assumed that a proportion of players are assigned a random strategy (ALLD or TFT), regardless of their traits. This makes inferences from traits to strategies far more difficult. As expected, we find that prejudiced strategies are particularly sensitive to this type of noise (Fig. 5). Prejudiced strategies are successful when the inferences drawn have at least some validity. For example, prejudiced players are good at learning that blue people tend to defect more frequently than yellow people. If, however, there exists no connection whatsoever between color and behavior, prejudiced players will nonetheless draw inferences–wrong ones–and apply them with equal confidence, with potentially disastrous results. As a result, higher levels of noise in the mapping from traits to strategies significantly lower the fitness of prejudiced strategies.

**Figure 6:**
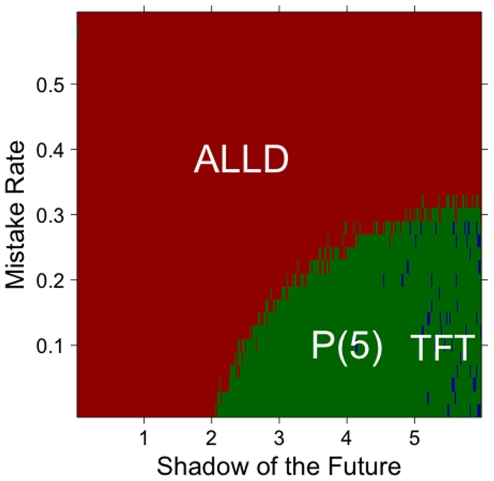
Strategies with the highest average score, as a function of the rate of mistake. The rate of mistake refers to the probability that a player will play randomly instead of following its strategy. The higher the mistake rate, the more the population plays cooperate or defect at random. Prejudiced strategies perform well for low levels of mistake and intermediate values of the shadow of the future. When more randomness is added, however, inferences drawn by prejudiced strategies become less and less reliable. The results are based on one hundred simulations with 50% TFT, 50% ALLD and one prejudiced player.

A second type of noise we investigated is the possibility that players play in discordance with their strategy. They might do so by mistake or strategically, but the result is that the strategy of these players becomes more difficult to determine for observers. More practically, we assume that in every time step of the game, a player plays a random move (cooperate or defect) with some probability, instead of the one she is programmed to play. For example, a TFT player might defect when she is meant to cooperate. Again, we find in this case that the larger the rate of mistake, the less competitive prejudiced strategies become (Fig. 6). Note that this type of noise also affects the efficiency of TFT for the same reasons: noise leads to wrong inferences about an individual's strategy, and hence to an ill-suited response in the next period [21].

**Figure 7:**
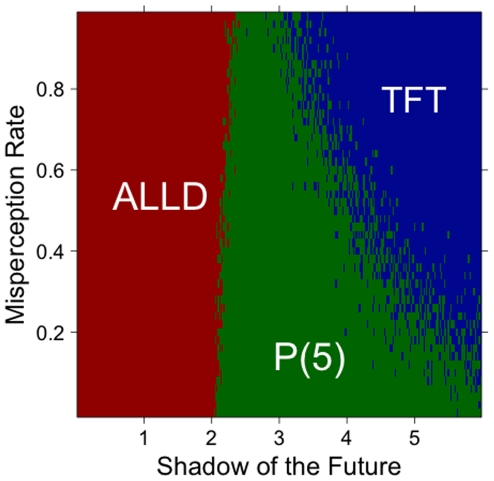
Strategies with the highest average score, as a function of the rate of misperception. The rate of misperception refers to the probability that a prejudiced player will record its opponent's attributes with some mistake. For example, a true sequence of traits 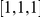 might be recorded as 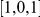. The higher the misperception rate, the more often a prejudiced player will wrongly record one of her opponent's traits. Here, the rate of misperception affects the range of shadow of the future for which prejudiced players (here P(5)) perform better than any other strategies. The results are based on one hundred simulations with 50% TFT, 50% ALLD and one prejudiced player.

A third type of noise involves perceptions. Suppose that prejudiced players might misperceive the traits of their opponents. For example, they might perceive them as rich when they are really poor, or as blue when they are red. We implemented this concept by assuming that, with some probability, a prejudiced player will misperceive one of his opponent's traits. For example, a true DNA sequence 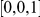 might instead be recorded by the prejudiced player as 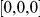. Again, we find that the results are damaging for prejudiced strategies, although not as much as for the other types of noise analyzed above (Fig. 7).

## Discussion

The outbreak of cooperation in a hostile environment such as the prisoner's dilemma has been the object of a large amount of research in numerous fields ranging from social sciences [15], [22], [23] to biology [24] and physics [25], [26]. Probably the most important finding in this literature is that TFT defeats most other strategies in a wide range of environments [15], [27]. However, we showed here that TFT suffers from one major drawback, which limits its applicability in the real world: it is relatively slow to learn [28]. Considering for example Axelrod's original setup, TFT players need to meet at least once with another player before they form an “opinion”of her. This strategy is well-suited if the population is relatively small and stable (no births or migration) because the initial risk of exploitation pays off, given the large expected number of interactions with the same players. However, when populations renew themselves rapidly, as is the case (to varying degrees) in bacterial, animal, or human populations, TFT can incur high initial costs because it cooperates in the first round against numerous defectors, yet without reaping the long-term benefits of this learning process. In fact, these costs are very high if the population is composed mostly of defectors. This can be particularly problematic when initial losses are difficult to make up for, as is the case for example when the rich get richer [29].

In other words, the problem with TFT is that it does not draw any inferences about the population. It treats every individual as unique. We showed here that this is often inefficient, and that strategies that extrapolate from a small number of attributes (prejudiced strategies) often perform better than both TFT or defect, because they allow for faster (though less accurate) learning.

These findings have clear empirical implications, in that they relate a population's speed of renewal–a function of, among other, a country's size, its openness, migration, birth and death rate–to the prevalence of prejudices in that population. One prediction in particular would be that people in societies traditionally characterized by low rates of migration (e.g., islands) would judge their peers on the basis of subtler cues that those in societies with high levels of migration. When the population evolves slowly, the expected number of interactions with a given person (the shadow of the future) is high, and hence investing in finer discrimination procedures pays off in the long run.
